# 

**Published:** 1921-09-03

**Authors:** 


### Birth Control.

Under the presidency of Dr. Marie C. Stopes, a Society has been formed having among its objects "to bring home to all the fundamental nature of the reforms involved in conscious and constructive control of conception," and "to supply all who still need it with the full knowledge of sound physiological methods of control." Membership is open to all who subscribe a minimum of 1s. per annum. The founder, Dr. Marie Stopes, has personally undertaken to guarantee any deficit for the first year. The Society is called the Society for Constructive Birth Control and Racial Progress, and its Hon. Secretary is Dr. Marie Stopes' husband, Councillor H. V. Roe. The General Executive Committee consists of five persons, apparently laymen and laywomen, and the Medical Research Committee includes Dr. Jane L. Hawthorne, Nurse Maud Hebbes, Mr. Geo. Jones, M.A. (barrister-at-law), and Mr. E. B. Turner, F.R.C.S., with power to add to their number.

### Beds for Tuberculosis.

Sir Alfred Mond has stated that the number of beds in institutions approved by the Ministry of Health increased from 8,888 (or .258 per 1,000 of the poulation) in August 1914 to 16,396 (or .470 per 1,000 of the population) in August 1920.

### Medical History of the War.

JUST before the adjournment of the House of Commons, Sir R. Sanders (for the Secretary of State for War) stated at question-time that the medical history of the war is being prepared in a series of volumes. The first volume of general history is practically ready for publication, and the first volumes of surgery and diseases of the war are practically ready for printing. The medical statistics of the war are being prepared by the Medical War Records Section, which is under the Minister of Pensions.

### The Coalfield Motor Ambulance Service.

THE Priory for Wales, which has now been approved as the representative of the Order of St. John in the Principality and Monmouthshire, is rapidly extending its work. The coalfield motor transport service has justified itself during the past three months, and used the ten cars to carry 500 cases. A new ambulance station has been opened at Tumble, in Carmarthenshire, and the colliery companies in the area have entered into a contract with the Priory. Not only does this branch of the Priory's activities claim the approval of the coalowners, but the miners' lodges also have sent delegates to the headquarters at Cardiff in support of the scheme. A number of depôts to provide médical comforts to the sick poor has likewise been established in various parts of North and South Wales.

### Welfare Schemes for Industrial Firms.

THE current number of the *Journal of Industrial Welfare* gives particulars of its organisation for the purpose of assisting business firms and industrial concerns in connection with problems and innovations concerning their *personnel*. Under the head of Preliminary, Personnel, and Development, it undertakes to assist firms from the inception of their welfare schemes to any form or forms that may subsequently ensue; it will undertake complete responsibility for carrying out welfare schemes, or will recommend adequately qualified managers, supervisors, or personal service directors. It has under consideration a training scheme for probationers which will include co-operation with social study schools organised by the Universities and other recognised bodies. It undertakes visits of advice, or will send speakers to explain the meaning and possibilities of welfare work. The accounts of the activities of the Industrial Welfare Society (51 Palace Street, S.W.) and its member firms will be published monthly in the *Journal*.

### Paying Patients at Southmead Infirmary.

THE Bristol Board of Guardians has adopted a scheme for the admission, at discretion, of paying patients at Southmead Infirmary at a weekly charge of £2 2s. or £3 3s., according to the size of the ward. Applicants must produce a recent certificate from a doctor who had been attending the case within a month of the application, stating the nature of the illness. The weekly charge includes attendance by the infirmary resident medical staff, with whom the patient's private doctor may confer. Where operations are required a fee will be paid to the surgeon at a râte specified in a table to be drawn up. The spare accommodation at Southmead is limited, but when beds are vacant they can be used for paying patients on the above terms.

### Are Cottage Hospitals Half Empty?

A CO-ORDINATION scheme is on foot to make a common arrangement between Salop Infirmary and the cottage hospitals of the county. The cottage hospitals are said to be often only half full, and the idea is to send to them, whenever there are vacant beds, patients who are not fit to return home and yet sufficiently out of danger to be liable to be discharged because of the number of cases waiting.

### The College of Nursing.
### (Limited by Guarantee.)

### EDINBURGH CENTRE.

(Hon. Secretary, Miss Cathcart, The Elms, Whitehouse Loan.)

College Endowment Fund Sale of Work, December 1, 1921.—The following is the complete list to date of those who have consented to receive contributions in money or in kind for the various stalls:—*City Hospital, Edinburgh*: Miss Thomas. *College Members*: Miss Cumming, R.R.C., Longmore Hospital; Miss Turnbull and Miss Cathcart, The Elms, Whitehouse Loan, Edinburgh. *Mental Hospitals*: Miss Davidson, Bangour Hospital; Miss Thyne, West House; Miss Wise, Craig House. *Nursing Homes and Private Nurses*: Miss McGarth, 26 Chalmers Street; Miss Graham, 15 Alva Street; Miss Walker, 7 Rutland Square. *Queen's Nurses*: Miss White, Q.V.J.I.N.S., 25 Castle Terrace, Edinburgh. *Royal Infirmary, Edinburgh*: Miss Gill, R.R.C., Royal Infirmary. *Tea-Room*: Miss Gordon, 8 Drumsheugh Gardens.

### SHEFFIELD CENTRE.

Hon. Secretary, Mrs. Barnes, 34 Wilkinson Street.

Saturday, October 8.—*Jumble Sale in the Lower Cutlers' Hall*.—The following matrons have kindly consented to receive parcels: Miss Earle, Royal Hospital; Miss Smeeton, Royal Infirmary; Miss Hollis, Children's Hospital; and Miss Hancox, Superintendent, Queen's Nurses, 334 Glossop Road—all of Sheffield. For Rotherham: Miss Buckle, Superintendent, Queen's Nurses, Highfield, Doncaster Road, Rotherham. Mrs. Watson, Hon. Treasurer, 426 Glossop Road, or Miss C. E. Abbott, 34 Bristol Road, Sheffield, will be pleased to arrange for the collection of large parcels on the receipt of a postcard. Helpers will be welcome at the Cutlers' Hall on the afternoon preceding the Sale from 3-7 p.m., and on the morning of the Sale at 10 a.m.

### CARDIFF CENTRE.

Hon. Sec., Miss M. E. Hitch, Anthony House, Newport Road.

Tuesday, September 6, 3-6.30 p.m.—Dr. and Mrs. Robinson, of Hillside, Penglan, Cardiff, will be at home to members of the Cardiff Centre.

Those members who have not already paid their subscriptions are asked to send them to Miss Forster-Feather, Hon. Treasurer, Royal Gwent Hospital, Newport (Mon.).

### "Our Day."

Sir Arthur Stanley, Chairman of the Joint Council of the British Red Cross Society and Order of St. John, appeals for helpers on "Our Day," which this year will be October 1. Ladies who are willing to sell flags are asked to send in their names to 19 Berkeley Street, W. 1, as soon as possible, indicating the district which they prefer. A great many will be needed, as the area to be covered extends from Kenley in the south to Potter's Bar in the north, from Erith in the east to Uxbridge and Staines in the west. Some of the leading features of the Joint Council's programme are the support of hospitals; help to the Central Council for Infant and Child Welfare; clinics no longer needed for pensioners to be used for civilians who are unable to pay the high fees usually charged; the hire of surgical and sick-room requisites for nominal sums; a motor-ambulance service throughout the country. The latter is already proving a great boon to residents in rural districts. In the short time it has been working over 60,000 patients have been carried upwards of 600,000 miles.

### University College Hospital Medical School.

The Goldsmid Entrance Exhibitions, of 112 guineas each, have been awarded to A. E. Blake Pritchard, King's College, Cambridge, and N. L. White, St. John's College, Cambridge.

### Matrons' and Sisters' Appointments.

St. George's Hospital, Bombay.—Miss Edith Macfarlane, R.R.C., has been appointed lady superintendent. She was trained at St. Bartholomew's Hospital, where she won the Clothworkers' Company prize, and was later matron of the Royal Ear Hospital. During the war she served in Malta, India, and Mesopotamia, and was awarded the Royal Red Cross, first class, with bar, and was mentioned in despatches.

Harrogate General Infirmary.—Miss Blodwen Thomas has been appointed night sister. She was trained at Cheltenham General Hospital, previously taking three years' training at the Jaffra Hospital, Birmingham; after her training at Cheltenham she was for six months staff nurse in the V.D. Department at St. Bartholomew's Hospital, E.C.

Sheffield Street Hospital, Kingsway.—Miss W. Madgwick has been appointed superintendent sister. She was trained at the London Temperance Hospital, and has been sister at St. Mary's Hospital, Paddington.

Norwich Isolation Hospital.—Miss D. Sandy and Miss A. Kitchen have been appointed sisters. The former was trained at the Portsmouth Infirmary and the Infectious Diseases Hospital, Portsmouth; the latter at Chelmsford General Hospital. Miss Kitchen has subsequently been staff nurse at the North-Eastern Fever Hospital, Tottenham, and at the Princess Mary's Hospital for Children, Margate.

Royal Edinburgh Asylum, West House.—Miss Elizabeth Cameron has been appointed assistant matron. She was trained at the Royal Asylum, Gartnavel, Glasgow, and at Dundee Royal Infirmary, where she held sister's post. She has been matron of the Lodge Auxiliary Hospital, Broughty Ferry, and has done war work in France.

Maltings Farm Sanatorium, Nayland.—Miss Ida J. E. Cordon has been appointed matron. She was trained at the London Hospital, where she was subsequently night sister. She has also worked as medical missionary in Delhi, India, done military nursing under the British Red Cross Society in France, and been matron of the East Anglian Sanatorium.

Victoria Hospital, Tientsin, North China.—Miss M. Lyle has been appointed matron, and also lady superintendent of the municipal nurses. Miss Lyle was trained at the Royal City of Dublin Hospital and the Rotunda Hospital. She served during the war with Queen Alexandra's Imperial Military Nursing Service in Egypt and on transport duty. She holds the certificate of the Central Midwives Board.

Middlesbrough Union Hospital, Holgate.—Miss E. B. Traynor has been appointed assistant superintendent nurse. She was trained at the Poor-Law Infirmary, Newcastle-on-Tyne, where she was later ward sister. She was for four years with the Territorial Force Nursing Service, serving first at the First Eastern General Hospital, Cambridge, and afterwards in France. Latterly she has been ward and maternity sister, and subsequently night superintendent of Holgate Hospital, Middlesbrough.

Fulwood Union Infirmary, Preston.—Miss J. M. Partridge has been appointed night superintendent nurse. She was trained at Crumpsall Infirmary, Manchester, where she was later sister and night superintendent; she has held the post of second assistant matron of the Withington Institution.

York County Hospital.—Miss Isabel Cameron has been appointed assistant matron. She was trained at the Victoria Infirmary, Glasgow, where she had charge later of the operating theatre. She held the posts of night sister, charge sister, and home sister at the 32nd British General Hospital, Amara, Mesopotamia, and has latterly been on the staff of the Scottish Association of Trained Nurses, Edinburgh. Miss Cameron holds the housekeeping diploma of the Edinburgh School of Cookery, and was assistant domestic superintendent at Edinburgh Royal Infirmary; she also holds the certificate of the Central Midwives Board.

September 3, 1921] [Price Twopence

### The Hospital

**"Our Day"** ... ... ... ... ... ... ... 392

**Matrons' and Sisters' Appointments** ... ... 392

**RATES OF SUBSCRIPTION** (post free, payable in advance).
Three months, 3/3; six months, 6/6; twelve months, 13/-. (Should the cost of printing and paper as well as increased postage, necessitate alteration of these rates, the period paid for will be reduced proportionately on unexpired subscriptions.) THE HOSPITAL "Index" to Volume LXIX., October 1920 to March 1921, is now ready, price 1/1 per copy, post free. Address The Manager, The Hospital, "The Hospital" Building, 28/29 Southampton Street, Strand, London, W.C. 2.

28 & 29 SOUTHAMPTON STREET,

